# Phylogeography and dispersal in the velvet gecko (*Oedura lesueurii*), and potential implications for conservation of an endangered snake (*Hoplocephalus bungaroides*)

**DOI:** 10.1186/1471-2148-12-67

**Published:** 2012-05-14

**Authors:** Sylvain Dubey, Benjamin Croak, David Pike, Jonathan Webb, Richard Shine

**Affiliations:** 1 Department of Ecology and Evolution, Biophore Bld, University of Lausanne, Lausanne 1015, Switzerland; 2 School of Biological Sciences, University of Sydney, Sydney, NSW 2006, Australia; 3 School of Marine and Tropical Biology, James Cook University, Townsville, QLD, Australia

**Keywords:** Australia, Phylogeography, Dispersal, Reptile, Landscape genetics, Conservation

## Abstract

**Background:**

To conserve critically endangered predators, we also need to conserve the prey species upon which they depend. Velvet geckos (*Oedura lesueurii*) are a primary prey for the endangered broad-headed snake (*Hoplocephalus bungaroides*), which is restricted to sandstone habitats in southeastern Australia. We sequenced the ND2 gene from 179 velvet geckos, to clarify the lizards’ phylogeographic history and landscape genetics. We also analysed 260 records from a longterm (3-year) capture-mark-recapture program at three sites, to evaluate dispersal rates of geckos as a function of locality, sex and body size.

**Results:**

The genetic analyses revealed three ancient lineages in the north, south and centre of the species’ current range. Estimates of gene flow suggest low dispersal rates, constrained by the availability of contiguous rocky habitat. Mark-recapture records confirm that these lizards are highly sedentary, with most animals moving < 30 m from their original capture site even over multi-year periods.

**Conclusion:**

The low vagility of these lizards suggests that they will be slow to colonise vacant habitat patches; and hence, efforts to restore degraded habitats for broad-headed snakes may need to include translocation of lizards.

## Background

To conserve an endangered species, we need to provide suitable habitat, shelter, prey items, and other resources (see e.g. [1-4]). Prey availability may be one of the most critical issues, especially for predators with specialized diets [5,6]. If management plans for endangered species include the restoration of habitat, we need to know if the endangered taxon itself is vagile enough to locate and colonise the newly-available sites. Evaluating the likelihood that significant prey species also will colonise restored areas is also important; if they do not do so (perhaps because of poor dispersal capacity), otherwise-suitable habitat may be unable to support populations of the endangered taxon.

The Broad-headed snake (*Hoplocephalus bungaroides*, Elapidae) is a small elapid snake restricted to rocky areas (sandstone plateaux) within a 200 km radius of Sydney, in south-eastern Australia [6]. These snakes were abundant at the time of European colonisation 200 years ago, but have now disappeared from most of its former range [7,8]. The threatening processes include habitat degradation and fragmentation resulting from the removal and destruction of critical shelter sites (especially, exfoliated rock that forms thermally-suitable retreat sites during the coldest parts of the year: [8]), forest overgrowth [3,4,9] and illegal collection of animals for the pet trade [10]. Efforts at habitat restoration have produced encouraging results, with the snakes and their lizard prey rapidly colonising sites by themselves where artificial rocks have replaced stolen natural rocks [11] and where trimming of vegetation has allowed increased sunlight penetration [3,4]. However, these studies have focused on sites very close to extant populations of snakes and their prey; the prospectus for successful colonisation of more distant sites remains unclear.

For relatively isolated habitat patches to be colonised, both the snakes and their prey must be able to reach them. Landscape-genetic analyses have confirmed that broad-headed snakes often move between adjacent outcrops (distance between outcrops: 0.9 to 10.7 km), and thus are likely to rapidly find any restored habitat patches [12]. The probability of colonisation by the snakes’ prey species has not been studied, and is the subject of the present paper. Broad-headed snakes consume a diversity of vertebrate prey taxa, but the most important taxon (especially during cooler months of the year, when the snakes are restricted to rock outcrops) is the velvet gecko (*Oedura lesueurii*, Diplodactylidae: [6]). Indeed, velvet geckos comprised 70% of prey items consumed by juvenile *H. bungaroides*[6]. Like *H. bungaroides, O. lesueurii* is restricted to rock outcrops [13,14]. The predator–prey interaction between these two taxa presumably has been a long-running one, because geckos from populations sympatric with this snake species are reported to display a suite of antipredator tactics not seen in conspecific geckos from populations allopatric to broad-headed snakes ( [15]; but see [16] for data that challenge this conclusion). Local coadaptation is likely only when gene flow is restricted between populations (e.g. [15,17,18]), allowing the evolution of spatial heterogeneity in relevant traits.

To evaluate the history of this predator–prey interaction, we need to know the timeline not only for the predator’s evolution [12] but also for the prey’s evolution (current study). Because *O. lesueurii* is an important prey species for *H. bungaroides*, we also need to evaluate the potential for *O. lesueurii* to colonise newly restored areas of rocky habitat*.* We can clarify this issue with a study of landscape genetics (e.g., what are the spatial scales of current and historical rates of gene flow?) and direct measures of dispersal, based on mark-recapture fieldwork.

## Results

### Phylogenetic analyses and molecular dating

**Figure 1:**
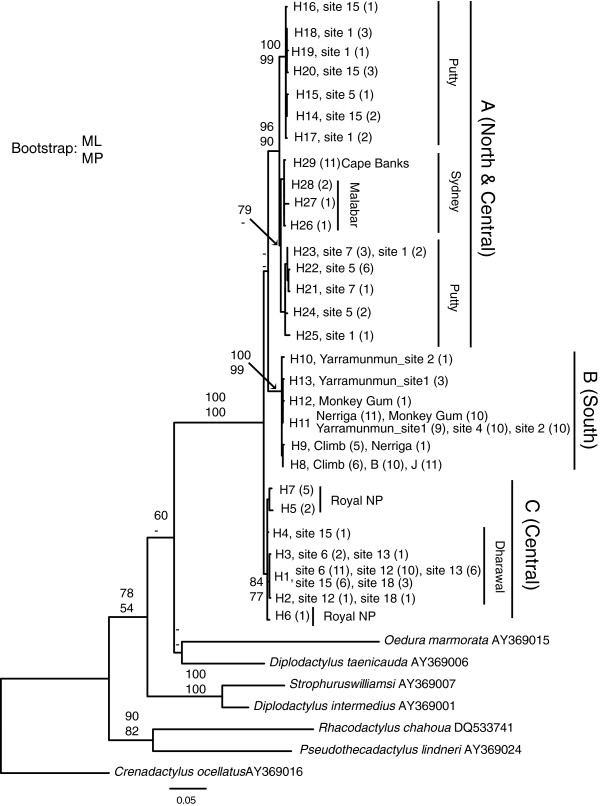
**Geographical distribution of the gecko (*Oedura lesueurii*) tissue samples used for genetic analyses, with mean ϕ**_**ST**_** values and K2P distance between lineages (A, B, and C).**

The 179 samples of *O. lesueurii* showed 29 haplotypes (H1-H29, [Genbank: JQ779339-JQ779366]) of 710 bp. The complete dataset included 369 variable sites of which 237 were parsimony-informative. As the two phylogenetic methods showed similar arrangements of the main branches, Figure 1 only shows the relationship between haplotypes for the ML analyses (see Additional file 1 for the MP tree). Three main lineages are present within the study area, the first (A) including populations from the north and central areas (Putty, Malabar, and Cape Banks; ML and MP analyses show bootstrap support of 96% an 90% respectively), the second (B) restricted to populations from the south (Morton; ML and MP analyses show bootstrap support of 100% and 99%), and the third (C) strictly central populations (Dharawal and Royal NP sites; ML and MP analyses show support of 84% and 77%). The mean K2P distance between the lineages was 3.7, 3.5, and 4.3% for A-C, B-C, and A-B respectively.

Dating analyses based on the secondary calibration points revealed a first divergence within *O. lesueurii* about 5.68 million years ago (Ma; 95% HPD: 2.73 – 10.76), with a split between haplotypes within lineages occurring 2.94 Ma (95% HPD: 1.21 – 5.18), 1.07 (95% HPD: 0.28-0.94), and 1.58 (95% HPD: 0.50 – 2.96) for A, B, and C respectively. Dating analyses based on a standard divergence rate of 1.3% (derived from numerous previous studies; see Methods section) gave similar results, with a first divergence within the species about 5.00 Ma (95% HPD: 2.88 – 8.06), with a split between haplotypes within lineages occurring 2.36 Ma (95% HPD: 1.30 – 3.96), 0.83 Ma (95% HPD: 0.30 – 1.83), and 1.25 Ma (95% HPD: 0.56 – 2.53) for A, B, and C respectively.

### Population and landscape genetic analyses

Overall, the ϕ_ST_ between populations varied from 0 to 1.0 (see Additional file 2, with a mean value of 0.81. The mean of the pairwise ϕ_ST_ value within each lineage was 0.62, 0.52, and 0.24 for A, B, C respectively.

Based on the Mantel and partial Mantel tests, the observed genetic structure (ϕ_ST_) in *Oedura lesueurii* populations was best predicted by a combination of distance and minimum elevation between sites (AIC value = −257.78; AIC weight = 0.27; R^2^ = 57.87; straight-line distance, partial corr.: 0.56; minimum elevation, partial corr.: -0.51; Table 1). The second-best model included the number of rivers, the minimum elevation and the distance between sites (AIC value = −257.72; AIC weight = 0.26; R^2^ = 58.85; rivers, partial corr.: -0.44; minimum elevation, partial corr.: -0.61; straight-line distance, partial corr.: 0.15). Three more models deviated from the best model by less than two units (i.e., ∆AIC < 2), and all these models include the minimum elevation and the straight-line distance between sites as explanatory variables, further indicating the importance of these parameters. Because the true distance between sites was less informative than the straight-line distance (straight-line distance, R^2^ = 31.48; true distance, R^2^ = 20.78), we used latter variable in our analyses (see Table 1).

**Table 1:** **Results of mantel and partial mantel test of landscape genetics of the gecko *Oedura lesueurii, *with a listing of variables included in the models (number of rivers [River] and number of roads [Walking track; Dirt Road; Paved Road; All road] between sites, minimum elevation between sites sites [min. elevation], mean elevation of sites minus the minimum elevation between sites [Mean elevation - min. elevation], straight-line distance [Distance] and true distance between sites [True distance]), the number of parameters per model, R**^**2**^**(total variance explained by the model), coefficient of correlation, P-value of parameters (The level of significance for our tests was set at α = 0.0028 (Bonferroni correction; i.e. 0.05/18 = 0.0028, where 18 represents the number of tests performed), AIC, Δ AIC, and AIC weight**

**Variable**	**K**	**R2**	**Coeff corr.**	**P-value**	**AIC**	**ΔAIC**	**AIC w**
**Distance & min. elevation**	**3**	**57.87**			**−257.78**	**/**	**0.2686**
**Distance**			**0.56**	0.0001*			
**Min. elevation**			**−0.51**	0.0001*			
River & Min. elevation & distance	4	58.85			−257.72	0.06	0.2606
River			−0.44	0.0001*			
Min. elevation			−0.61	0.0001*			
Distance			0.15	0.0362			
River & Dirt road & Distance & Min. Elevation	5	59.13			−256.29	1.49	0.1275
River			−0.44	0.0001*			
Dirt road			0.19	0.0085			
Distance			0.37	0.0001*			
Min. elevation			−0.47	0.0001*			
Min. elevation & Dirt road & distance	4	58.08			−256.20	1.58	0.1219
Min. elevation			−0.75	0.0001*			
Dirt road			0.15	0.0465			
Distance			0.06	0.3982			
Distance & mean - min. elevation & min. elevation	4	57.87			−255.79	1.99	0.0993
Min. elevation			−0.75	0.0001*			
Mean - min. elevation			0.05	0.4932			
Distance			0.14	0.0516			
Min. elevation	2	55.59	−0.75	0.0001*	−255.44	2.34	0.0834
Min. elevation & mean - min. elevation		55.84			−253.91	3.87	0.0388
Min. elevation	3		−0.75	0.0001*			
Mean - min. elevation			0.05	0.4870			
Distance & mean elevation - min. elevation	3	39.71			−228.22	29.56	0.0000
Distance			0.56	0.0001*			
Mean elevation - min. elevation			0.29	0.0002*			
Distance & river	3	36.65			−224.13	33.65	0.0000
Distance			0.56	0.0001*			
River			−0.23	0.0019*			
Distance & dirt road	3	31.49			−217.67	40.11	0.0000
Distance			0.56	0.0001*			
Dirt road			0.01	0.9289			

Only Mantel and partial Mantel tests (total of 18) with the 10 best AIC values are shown.

In grey, best model (bold) and models with a Δ AIC < 2.

**Figure 2:**
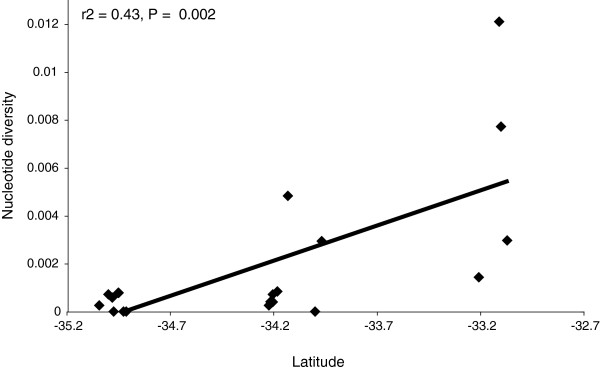
**Phylogeny of the ND2 fragment of the gecko *Oedura lesueurii *in southeastern Australia, analysed using a maximum likelihood procedure.** Values in branches are indices of support for the major branches for maximum likelihood (ML) and maximum parsimony (MP) analyses (percentage of 1000 replications for ML and MP).

The nucleotide diversity at a site tended to increase with latitude (i.e., was higher at more northern sites; F_1,19_ = 13.88, P = 0.002*; Figure 2), and we did not detect any significant relationships between nucleotide diversity and the number of samples, the elevation, or the longitude of sites.

The samova revealed high *F*_CT_*(*among population groups) values for all the groups and small *F*_SC_*(*within population group) values in cases of 9 to 19 groups, indicating very high population structure. For example, at *K* = 9 the majority of variation (94.32%) is among groups, although 0.03% of variation at the level of among populations within groups still represents highly significant population structuring in the remaining population groups (*P* < 0.001). At *K* = 2, the two clusters identified were the populations of lineage B (Morton) vs lineages A (Putty, Malabar, and Cape Banks) and C (Dharawal and Royal NP), and at K = 3, the three clusters were the populations of lineage A, B, and C.

### Dispersal distances of free-ranging geckos

**Figure 3:**
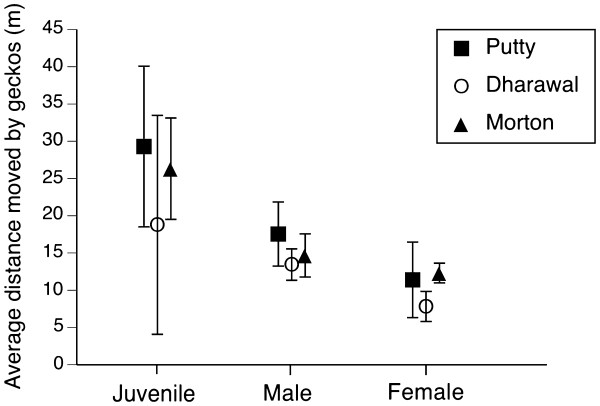
**Relationship between the latitude of a site, and the nucleotide diversity of the gecko *Oedura lesueurii *within that site.**

In total, we obtained records of the distances moved by 260 geckos, over time periods ranging from 24 to 928 days between recaptures (average time between recaptures = 203.1 days). We used ANOVA to compare gecko movements among regions and between sexes and age classes over time. There were no significant interaction effects, so we only describe main effects. *Oedura lesueurii* was highly sedentary. Marked lizards did not tend to move further away from their initial capture site with increasing time (F_2,252_ = 2.08, P = 0.15), indicating that they have fixed home ranges. There was no difference in mean movement distances between male, female or juvenile geckos (F_2,252_ = 1.84, P = 0.16), and the average distances moved were less than 30 m (Figure 3). The maximum dispersal distances recorded were 1.648 km for an adult male, 1.442 km for an adult female, and 1.577 km for a juvenile.

## Discussion

**Figure 4:**
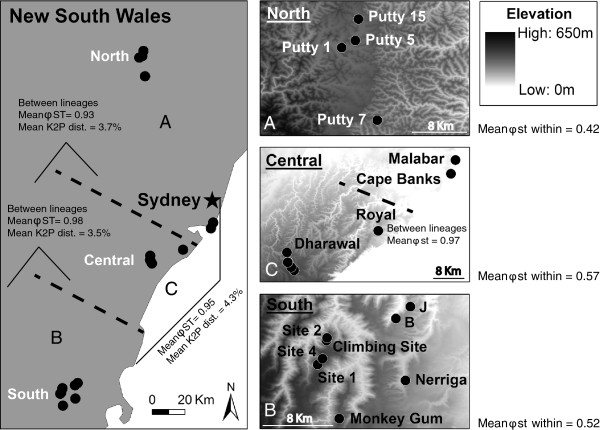
**Average distances moved by juvenile, male and female geckos * Oedura lesueurii *at sites in the Putty region (Yengo and Wollemi National Parks), Dharawal State Conservation area and Morton National Park.** Mean recapture intervals differed among samples, but averaged 203 days. The graph shows mean values and associated standard errors.

Our study revealed ancient genetic divergences within *Oedura lesueurii* from southeastern Australia, beginning in the Miocene-Pliocene (5.68 – 5.0 Ma) and resulting in three geographically well-defined lineages (North, Central, and South; Figures 4,1). Similarly, our population genetics analyses showed a strong spatial structure among our 20 populations as well as within lineages, with a lack of haplotype sharing between populations separated by only 3.7 km (ϕ_ST_ = 0.70). In addition, our landscape genetic analyses identified distance as the major barrier to gene flow (ϕ_ST_) between populations. In contrast, an absence of areas with low elevation between sites (e.g. the absence of deep valleys separating populations) favoured dispersal. In this case, areas of high elevation between sites reflects continuous favourable habitat (e.g. rocky outcrops). Similarly, gene flow in the broad-headed snake *H. bungaroides* mostly occurs along sandstone plateaux rather than across the densely forested valleys that separate plateaux [12]. Consistent with these genetic analyses, our field data (5 years of mark-recapture studies) revealed that *O. lesueurii* are sedentary. Marked individuals typically remained within close proximity (tens of metres) to their original capture site for years, consistent with earlier reports that some females return to their natal sites to lay eggs [14]. Previous phylogenetic studies on southeastern Australian reptiles (e.g. [19,20]) have revealed similar ancient splits between populations.

In both of these taxa, a southern lineage (restricted to Morton NP) differs significantly from conspecifics in the Sydney area. Sumner et al. [20] suggested that the break between the southern and northern clade of *H. bungaroides* occurs in a geologically distinctive area where volcanic soils cover the sandstone plateaux [21], acting as a barrier to gene flow. The same may be true of other sandstone specialist species such as *O. lesueurii*. The strong genetic structure observed in this study is consistent with general patterns observed in various taxa distributed in eastern Australia [22] and could be attributed to the ancestral position of the mesic biome (which dominates eastern Australia), and hence allowed localized endemism from long term persistence of populations through multiple climatic cycles [22]. Finally, the observed gradient of genetic diversity in *O. lesueurii* throughout the study area (decreasing diversity with increasing latitude) may be the result of harsher historical conditions in the southern part of the range (Last Glacial Maximum; [23]). The species reaches its current southern distributional limit close to our study sites in Morton NP [24].

Overall, the diversification of Australian geckos is ancient and may have originated from a Gondwanan vicariance (e.g. about 70 Ma for the diplodactyloids: Oliver and Sanders, 2009). In this respect the geckos differ from most other squamates, which colonized Australia from Asia more recently (e.g. [25-28]). Similarly, Australian geckos show relatively ancient intraspecific diversification (see e.g. [29-32]; this study). The diversification of at least one of the gecko’s major predators (the broad-headed snake *H. bungaroides*) is much more recent, as the split between the genera *Hoplocephalus* and *Paroplocephalus* occurred less than 3 Ma [26], and the oldest split between *H. bungaroides* lineages about 0.8 Ma [20]. Consequently, *O. lesueurii* was established across much of its current range in southeastern Australia long before the evolutionary origin of *H. bungaroides.* Our results support the plausibility of the conditions required for natural selection to produce adaptive local differentiation in geckos: that is, genetic variation among populations and low gene flow between them [33,34].

## Conclusions

From a conservation perspective, the low dispersal rates of *O. lesueurii* have two main implications. The first is that this gecko will be slow to recolonise any local areas from which it is extirpated (perhaps by chance abiotic events, predators, or human disturbance). Thus, habitat suitability for the endangered broad-headed snake may be spatially heterogeneous as a result of relatively ancient local events that reduced gecko numbers. Second, the low dispersal rates of the geckos need to be considered in any management plan that includes the restoration of degraded habitat previously hosting *H. bungaroides*. The poor dispersal capacity of *O. lesueurii* (unlike *H. bungaroides* itself; [12]) likely will delay or prevent natural recolonisation of geckos in restored areas, unless those areas are very close to extant populations. Consequently, we may need to reintroduce *O. leseurii* to such areas in order to guarantee successful habitat restoration for *H. bungaroides*.

## Methods

### Tissue collection

We collected tissue samples from 179 geckos representing 20 populations in southeastern Australia (see Figure 4 and Table 2), by turning rocks and capturing animals by hand. Tissues were collected by toe-clipping, or from voluntary tail autotomy. Tissue samples were placed in 100% ethanol, transported to the laboratory and stored below 0°C prior to processing.

**Table 2:** **Number of tissue samples of the gecko *Oedura lesueurii, *and the longitude, latitude, and elevation, length and width of the collecting site, the number of samples and of haplotypes, and the nucleotide diversity at that site**

**Sites**	**Long.**	**Lat.**	**Elevation (m)**	**Length (m)**	**Width (m)**	**# samples**	**# hapl.**	**Nuc div.**
Dharawal Site 6_	150.8739	−34.2025	334	93	44	13	2	0.000403
Dharawal Site 12	150.8873	−34.2213	439	71	60	11	2	0.00026
Dharawal Site 13	150.8807	−34.2139	382	73	60	7	2	0.000408
Dharawal Site 15	150.8709	−34.1801	268	234	191	7	2	0.00084
Dharawal Site 18	150.8726	−34.2027	335	105	76	4	2	0.000714
Climb_Morton	150.3770	−34.9505	393	340	20	11	2	0.000779
Nerrigera_Morton	150.4695	−34.9973	201	800	50	12	2	0.000716
B_Morton	150.4583	−34.9248	234	172	25	10	1	0
J_Morton	150.4752	−34.9109	211	187	15	11	1	0
MonkeyGum	150.3919	−35.0420	367	5000	10	11	2	0.00026
Yarramunmun site 1	150.3666	−34.9788	395	1100	50	12	2	0.000584
Yarramunmun site 4	150.3724	−34.9719	392	700	50	10	1	0
Yarramunmun site 2	150.3777	−34.9475	395	1200	50	11	2	0.000781
Putty_site 7	150.7251	−33.2068	271	216	97	4	2	0.001433
Putty_site 15	150.6998	−33.0697	266	77	30	5	3	0.002873
Putty_site 5	150.6954	−33.0987	325	178	40	9	3	0.007716
Putty_site 1	150.6769	−33.1084	294	241	42	8	5	0.012807
Malabar	151.2602	−33.9666	15	350	80	4	3	0.002933
Cape Banks	151.2496	−33.9983	15	950	60	11	1	0
Royal NP	151.0816	−34.1297	117	400	10	8	3	0.004823

### DNA extraction and PCR amplification

We placed tissues in 200 mL of 5% Chelex containing 0.2 mg/mL of proteinase K, incubated them overnight at 56°C, boiled them at 100°C for 10 min, and centrifuged them at 13,300 g for 10 min. The supernatant, containing purified DNA, was then removed and stored at −20°C.

Double-stranded DNA amplifications of NADH dehydrogenase 2 (ND2) were performed with the primer pairs AT4882 (5’caacatgacaaaaattrgcccc 3’; see [35])/ND2R2 (5’ ratctaggaggccttakc 3’; specifically designed for this study). Amplification conditions included a hot start denaturation of 95°C for 3 min, followed by 35 cycles of 95°C for 1 min, 55°C annealing temperature for 1 min, 72°C for 1 minute 45 seconds. We then performed a final extension of 72°C for 7 min and visualized the sequence reactions on a 3730 xl DNA Analyzer (Applied Biosystems, CA, USA).

#### 2.3 Phylogenetic analyses

We aligned sequences using BioEdit [36] and assessed them by eye. A sequence of *Crenadactylus ocellatus* ([GenBank:AY369016]; the basal species of the Diplodactylidae according to [28]) was used to root the tree. Additional sequences of Diplodactylidae were included in the analyses: *Pseudothecadactylus lindneri* [GenBank:AY369024], *Rhacodactylus chahoua* [GenBank:DQ533741], *Oedura marmorata* [GenBank:AY369015], *Diplodactylus taenicauda* [GenBank:AY369006], *Diplodactylus intermedius* [GenBank:AY369001], and *Strophurus williamsi* [GenBank:AY369007].

We performed ML heuristic searches and bootstrap analyses (1000 replicates) with phyml [37] and we selected the model of DNA substitution using jModelTest 0.1.1 [37,38]. The HKY + G model [39] best fitted the dataset with a Bayesian Information Criterion (BIC; [40]). Finally, we used Paup* 4.0b10 [41] to perform maximum parsimony (MP) analyses using 100 random additions of sequences followed by tree bisection and reconnection branch swapping, and retaining at most 100 trees at each replicate. We estimated branch support using 1000 bootstrap replicates with the same heuristic settings.

### Population and landscape genetic analyses

We estimated population structure between all sites sampled by calculating ϕ_ST_, taking into account haplotype frequencies and the genetic distance between haplotypes, in Arlequin 3.0 [42]. We used the Kimura two-parameter genetic distance (K2P; [43]) as our genetic model.

We performed Mantel and partial Mantel tests [44] using the software fstat Version 2.9.3.2 [45], with genetic distance as the dependent variable. The independent variables were the number of intervening rivers (River; i.e. the number of rivers crossing the strait-line distance between two locations) and roads (Walking track; Dirt Road; Paved Road; All roads) between sites, the minimum elevation between sites, the mean elevation of sites minus the minimum elevation between sites, the straight-line distance and true distance between site (i.e., by calculating the surface length of a line connecting each pair of sites while incorporating an underlying digital elevation model at a resolution of 25 m; implemented using the 3D Analyst Tool in ArcMap 9.3, 9). P-values were calculated after 10,000 randomizations. The level of significance for our tests was set at α = 0.0028 (Bonferroni correction; i.e. 0.05/18 = 0.0028, where 18 represents the number of tests performed). Based on the results of the Mantel and partial Mantel tests, we selected the best model using Akaike’s information criterion (AIC; [46]; based on the variance of the residuals). We compared each candidate model based on its AIC scores and weights. The best supported models are those with high Akaike weights, and that deviate from the best model by less than two units (i.e., ∆AIC < 2; [47]).

We used the program samova 1.0 [48] to characterise population structure and to define groups of populations using genetic criteria. Given an *a priori* number of clusters (*K*), the software uses a simulated annealing procedure to define the cluster composition in which populations within a cluster are as genetically homogeneous as possible (*F*_SC_ minimised) and clusters are maximally differentiated from each other (*F*_CT_ maximised; [48]). The analysis was run for *K* = 2 to *K* = 19 and the significance of fixation indices was tested by 1023 permutations.

### Molecular dating

We performed dating analyses using Beast 1.6.2 [49] with an uncorrelated lognormal relaxed clock and a coalescent tree prior. The coefficient of variation frequency histogram did not abut against zero, meaning that there was among-branch rate heterogeneity within our data [50]. Consequently, as suggested by Drummond et al. [50], we used a relaxed molecular clock.

We used two secondary calibration points from a robust phylogeny focusing on Australasian geckos [28]: (1) The oldest split within the Diplodactylidae (i.e. between *Crenadactylus ocellatus* and the other members of the family: 66.2 Ma [95% HPD: 46.6-87.0]) and (2) the split between *Pseudothecadactylus* and the New Caledonian *Rhacodactylus chahoua* and the remaining members of the Diplodactylidae (60.3 Ma [95% HPD: 41.5-79.2]).

The analysis was performed with two independent chains and 20 million generations; chains were sampled every 1000 generations with a burn-in of 2 million generations. Additional simulations were run with the same dataset and the same models, but strictly based on a rate of divergence of 1.3% derived from numerous studies as e.g. Zamudio & Greene’s [51] study on snake mtDNA and from Macey’s et al. ( [52]; also used in e.g. [35,53,54]) work on lizards.

### Dispersal distances of free-ranging geckos

We conducted mark-recapture surveys on velvet geckos by turning rocks and measuring, individually marking (by toe-clipping) and releasing any geckos found. These studies were conducted in and around Morton National Park (Morton) on a monthly basis between March 2007 and October 2009, and in Dharawal Conservation area (Dharawal) and Yengo and Wollemi National Park (collectively, Putty) from March 2008 until November 2010. We classified geckos as adult males if they were > 40 mm snout-vent length (SVL) with overt hemipenial bulges; adult females if they were > 40 mm SVL and without such bulges; and juveniles if they were < 40 mm SVL. We determined the distance between rocks used by individual *O. lesueurii* using GPS co-ordinates imported into ArcGIS 10.0 [55].

## Competing interests

The authors declare that they have no competing interests.

## Authors’ contributions

SD, BC, DP, JW and RS contributed with the conceptual development of the work and the writing of the manuscript. SD, DP, BC, and JW collected samples and data in the field. SD, BC, DP carried out the analyses. All authors read and approved the final version of the manuscript.

## Supplementary Material

Additional file 1 Maximum parsimony (MP) consensus tree (50% majority rule). Click here for file

Additional file 2 ** Pairwise *ΦST *values *between populations *and p*-values***. Click here for file
