# Supplementary figures and images for: Phylogeography and dispersal in the velvet gecko (Oedura lesueurii), and potential implications for conservation of an endangered snake (Hoplocephalus bungaroides)
Source: BMC Evol Biol. 2012 May 14;12:67. doi: 10.1186/1471-2148-12-67 (PMC3494511; doi:10.1186/1471-2148-12-67)

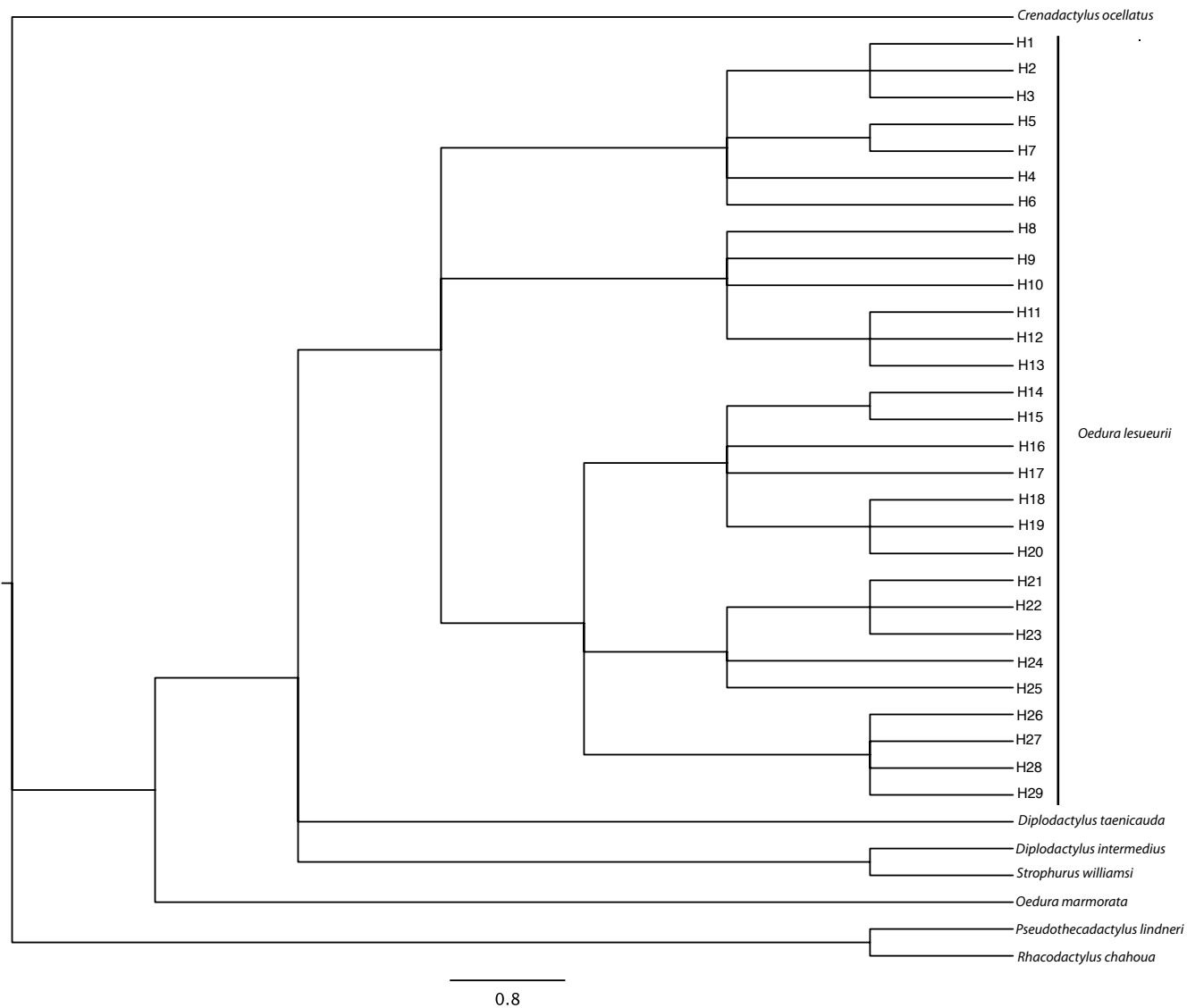

Supplement: Additional file 1 — Maximum parsimony (MP) consensus tree (50% majority rule). [file 1471-2148-12-67-S1.pdf]
