# Supplementary material for: Phylogeography and dispersal in the velvet gecko (Oedura lesueurii), and potential implications for conservation of an endangered snake (Hoplocephalus bungaroides)
Source: BMC Evol Biol. 2012 May 14;12:67. doi: 10.1186/1471-2148-12-67 (PMC3494511; doi:10.1186/1471-2148-12-67)
Supplement: Additional file 2 — Pairwise ΦST values between populations and p-values. [file 1471-2148-12-67-S2.pdf]

Population name

1	Dharawal Site 6
2	Dharawal Site 12
3	Dharawal Site 13
4	Dharawal Site 15
5	Dharawal Site 18
6	Climb_Morton
7	Nerrigera_Morton
8	B_Morton
9	J_Morton
10	MonkeyGum
11	Yarramunmun site 1
12	Yarramunmun site 4
13	Yarramunmun site 2
14	Putty_site 7
15	Putty_site 15
16	Putty_site 5
17	Putty_site 1
18	Malabar
19	Cape Banks
20	Royal NP

Population pairwise ΦSTs

	1	2	3	4	5	6	7	8	9	10	11	12	13	14	15	16	17	18	19
1	0																		
2	0.0487	0																	
3	-0.12292	0.01289	0																
4	0.05717	0.03178	0.00014	0															
5	0.09203	-0.07677	0.03464	-0.00769	0														
6	0.98266	0.98427	0.98068	0.97573	0.97704	0													
7	0.98302	0.98464	0.98135	0.97659	0.97811	0.77283	0												
8	0.99291	0.99577	0.99496	0.98978	0.9945	0.38433	0.85784	0											
9	0.99323	0.99598	0.99527	0.99041	0.99492	0.4	0.86385	0	0										
10	0.98963	0.992	0.99031	0.9854	0.98883	0.85749	-0.00346	0.95439	0.95659	0									
11	0.98507	0.98687	0.98409	0.9794	0.98141	0.82502	0.09111	0.90093	0.90528	0.12915	0								
12	0.99291	0.99577	0.99496	0.98978	0.9945	0.88386	-0.01617	1	1	-0.00917	0.15691	0							
13	0.98234	0.98397	0.98032	0.97527	0.9766	0.78592	-0.02946	0.8633	0.8694	0.0002	0.08868	-0.00917	0						
14	0.98405	0.98607	0.98041	0.97304	0.97215	0.97857	0.97968	0.99159	0.99223	0.98755	0.98187	0.99159	0.97825	0					
15	0.96303	0.96284	0.95028	0.94325	0.93374	0.96574	0.9673	0.97552	0.97712	0.97322	0.96901	0.97552	0.96527	0.88693	0				
16	0.9057	0.89924	0.87363	0.86732	0.84351	0.91026	0.91345	0.9145	0.91893	0.91564	0.9142	0.9145	0.90905	0.10653	0.68306	0			
17	0.85051	0.83928	0.80073	0.79481	0.75595	0.86664	0.87119	0.86786	0.87445	0.87145	0.87141	0.86786	0.86487	0.44358	0.14542	0.34559	0		
18	0.97185	0.97236	0.96091	0.95255	0.94356	0.96328	0.96449	0.97711	0.97884	0.97295	0.96681	0.97662	0.96196	0.83845	0.83115	0.59135	0.4771	0	
19	0.99381	0.99633	0.99568	0.99125	0.99536	0.9896	0.98983	1	1	0.99648	0.99159	1	0.98941	0.97667	0.93927	0.76225	0.65061	0.70421	0
20	0.75756	0.74654	0.68915	0.65156	0.62319	0.92805	0.93129	0.9367	0.94024	0.93665	0.93422	0.93741	0.92763	0.90875	0.89236	0.83728	0.78222	0.88457	0.94639

ΦST p-values

	1	2	3	4	5	6	7	8	9	10	11	12	13	14	15	16	17	18	19
1	*																		
2	0.36036+0.0606	*																	
3	0.99099+0.0030	0.48649+0.0562	*																
4	0.27928+0.0370	0.33333+0.0451	0.53153+0.0345	*															
5	0.19820+0.0353	0.99099+0.0030	0.33333+0.0333	0.68468+0.0279	*														
6	0.00000+0.0000	0.00000+0.0000	0.00000+0.0000	0.00000+0.0000	0.00000+0.0000	*													
7	0.00000+0.0000	0.00000+0.0000	0.00000+0.0000	0.00000+0.0000	0.00000+0.0000	0.00000+0.0000	*												
8	0.00000+0.0000	0.00000+0.0000	0.00000+0.0000	0.00000+0.0000	0.00901+0.0091	0.03604+0.0148	0.00000+0.0000	*											
9	0.00000+0.0000	0.00000+0.0000	0.00000+0.0000	0.00000+0.0000	0.00000+0.0000	0.00000+0.0000	0.00000+0.0000	0.99099+0.0030	*										
10	0.00000+0.0000	0.00000+0.0000	0.00000+0.0000	0.00000+0.0000	0.00000+0.0000	0.00000+0.0000	0.72072+0.0470	0.00000+0.0000	0.00000+0.0000	*									
11	0.00000+0.0000	0.00000+0.0000	0.00000+0.0000	0.00000+0.0000	0.00000+0.0000	0.00000+0.0000	0.14414+0.0337	0.00000+0.0000	0.00000+0.0000	0.17117+0.0550	*								
12	0.00000+0.0000	0.00000+0.0000	0.00000+0.0000	0.00000+0.0000	0.00901+0.0091	0.00000+0.0000	0.99099+0.0030	0.00000+0.0000	0.00000+0.0000	0.99099+0.0030	0.18919+0.0344	*							
13	0.00000+0.0000	0.00000+0.0000	0.00000+0.0000	0.00000+0.0000	0.00000+0.0000	0.00000+0.0000	0.99099+0.0030	0.00000+0.0000	0.00000+0.0000	0.45946+0.0656	0.14414+0.0337	0.99099+0.0030	*						
14	0.00000+0.0000	0.00901+0.0091	0.00000+0.0000	0.00000+0.0000	0.02703+0.0139	0.00000+0.0000	0.00000+0.0000	0.00000+0.0000	0.00000+0.0000	0.00000+0.0000	0.00000+0.0000	0.00901+0.0091	0.00000+0.0000	*					
15	0.00000+0.0000	0.00000+0.0000	0.00000+0.0000	0.00000+0.0000	0.00000+0.0000	0.00000+0.0000	0.00000+0.0000	0.00000+0.0000	0.00000+0.0000	0.00901+0.0091	0.00000+0.0000	0.00000+0.0000	0.00000+0.0000	0.00901+0.0091	*				
16	0.00000+0.0000	0.00000+0.0000	0.00000+0.0000	0.00000+0.0000	0.00000+0.0000	0.00000+0.0000	0.00000+0.0000	0.00000+0.0000	0.00000+0.0000	0.00000+0.0000	0.00000+0.0000	0.00000+0.0000	0.00000+0.0000	0.15315+0.0333	0.00000+0.0000	*			
17	0.00000+0.0000	0.00000+0.0000	0.00000+0.0000	0.00000+0.0000	0.00000+0.0000	0.00000+0.0000	0.00000+0.0000	0.00000+0.0000	0.00000+0.0000	0.00000+0.0000	0.00000+0.0000	0.00000+0.0000	0.00000+0.0000	0.00901+0.0091	0.11712+0.0385	0.00901+0.0091	*		
18	0.00000+0.0000	0.00000+0.0000	0.01802+0.0121	0.00901+0.0091	0.01802+0.0182	0.00000+0.0000	0.00000+0.0000	0.00000+0.0000	0.00901+0.0091	0.00000+0.0000	0.00000+0.0000	0.00000+0.0000	0.00000+0.0000	0.00901+0.0091	0.00901+0.0091	0.00000+0.0000	0.00000+0.0000	*	
19	0.00000+0.0000	0.00000+0.0000	0.00000+0.0000	0.00000+0.0000	0.00000+0.0000	0.00000+0.0000	0.00000+0.0000	0.00000+0.0000	0.00000+0.0000	0.00000+0.0000	0.00000+0.0000	0.00000+0.0000	0.00000+0.0000	0.00000+0.0000	0.00000+0.0000	0.00000+0.0000	0.00000+0.0000	0.00000+0.0000	*
20	0.00000+0.0000	0.00000+0.0000	0.00000+0.0000	0.00000+0.0000	0.00000+0.0000	0.00000+0.0000	0.00000+0.0000	0.00000+0.0000	0.00000+0.0000	0.00000+0.0000	0.00000+0.0000	0.00000+0.0000	0.00000+0.0000	0.00000+0.0000	0.00000+0.0000	0.00000+0.0000	0.00000+0.0000	0.00000+0.0000	0.00000+0.0000

Significance level (after Bonferroni correction) = 0.0002
